# Defining the Thrombotic Risk in Patients with Myeloproliferative Neoplasms

**DOI:** 10.1100/tsw.2011.108

**Published:** 2011-05-26

**Authors:** Fabrizio Vianello, Anna Battisti, Giuseppe Cella, Marina Marchetti, Anna Falanga

**Affiliations:** ^1^ Hematology and Clinical Immunology Unit, Thoracic, and Vascular Sciences Department, Padova University School of Medicine, Padova, Italy; ^2^ Cardiac, Thoracic, and Vascular Sciences Department, Padova University School of Medicine, Padova, Italy; ^3^ Division of Immunohematology and Transfusion Medicine, Ospedali Riuniti di Bergamo, Italy

**Keywords:** myeloproliferative neoplasms, essential thrombocythemia, polycythemia vera, thromboembolism, tissue factor, selectins, nitric oxide

## Abstract

Polycythemia vera (PV) and essential thrombocythemia (ET) are two Philadelphia-negative myeloproliferative neoplasms (MPN) associated with an acquired mutation in the *JAK2* tyrosine kinase gene. There is a rare incidence of progression to myelofibrosis and myeloid metaplasia in both disorders, which may or may not precede transformation to acute myeloid leukemia, but thrombosis is the main cause of morbidity and mortality. The pathophysiology of thrombosis in patients with MPN is complex. Traditionally, abnormalities of platelet number and function have been claimed as the main players, but increased dynamic interactions between platelets, leukocytes, and the endothelium do probably represent a fundamental interplay in generating a thrombophilic state. In addition, endothelial dysfunction, a well-known risk factor for vascular disease, may play a role in the thrombotic risk of patients with PV and ET. The identification of plasma markers translating the hemostatic imbalance in patients with PV and ET would be extremely helpful in order to define the subgroup of patients with a significant clinical risk of thrombosis.

## INTRODUCTION

Polycythemia vera (PV) and essential thrombocythemia (ET) are clonally derived malignancies grouped in the myeloproliferative neoplasms (MPN)[1]. They share characteristic features of hematopoietic progenitor cell proliferation leading to overproduction of one or more blood cell components. The cardinal features of these disorders are an increased red cell mass in PV and a high platelet count in ET. The clinical course of PV and ET is marked by variable incidence of progression to myelofibrosis and, to a lesser extent, to acute myeloid leukemia.

Specific molecular lesions characterize MPN, in particular the mutation JAK2V617F. This recent discovery has advanced our knowledge and potentially opened new avenues to the development of therapeutic tools.

Both PV and ET are associated with a well-defined hemostatic imbalance resulting in increased risk of both thrombotic and hemorrhagic episodes. In particular, arterial and venous thromboembolism is the major cause of morbidity and mortality in these patients (approximately 40%). This review will focus on recent advances in the pathogenesis of thrombosis in MPN, with particular interest in the role of new biological markers.

## THROMBOHEMORRHAGIC COMPLICATIONS IN PV AND ET

Evolution of PV or ET to myelofibrosis and transformation to acute myeloid leukemia represent the most relevant events in the course of these diseases, but bleeding and, more importantly, fatal thrombotic events are the leading cause of morbidity and mortality in PV and ET[2].

Hemorrhagic diathesis is more rare in patients with PV and ET, and it mostly affects cases with a very high platelet count (i.e., platelets >1,000 × $10^9$/L). An altered degradation and function of von Willebrand factor (vWF) has been shown to play a role[3]. The hemorrhagic diathesis observed in PV and ET recalls bleeding observed in quantitative as well as qualitative platelet defects. Common manifestations, in fact, are represented by easy bruising, epistaxis, and gingival bleeding[4].

Thromboses are both arterial and venous, and may occur virtually in any area. Arterial thrombosis, including acute myocardial infarction, cerebrovascular ischemic episodes, and peripheral arterial occlusion, represent about 60% of vascular events. Patients with PV experience more deep vein thrombosis and pulmonary embolism compared to those with ET. There is also a high prevalence of rare thrombosis, such as abdominal vein thrombosis, including extrahepatic portal vein occlusion, Budd-Chiari syndrome, and mesenteric vein thrombosis[5,6].

In a recent case-control study, the overall risk of thrombotic episodes was 6.6% per patient-year in a historical cohort of 100 patients with ET compared to 1.2% per patient-year in the control group. The rate of major hemorrhagic complications was 0.33% per patient-year[7].

## PATHOGENESIS OF THROMBOSIS IN MPN

Multiple factors are likely to contribute to the pathogenesis of thrombosis, including increased cell mass (in PV), platelet and leukocyte number, activation of platelets[8] and leukocytes, and their interaction to form platelet-leukocyte aggregates, in addition to prothrombotic circulating and endothelial factors. Even in the absence of manifest thrombosis, MPN patients present with a hypercoagulable state characterized by an increased concentration of several plasma markers of hemostatic system activation. Among the different mechanisms proposed is the clotting activation induced by the increased count, and the activation status of circulating platelets, erythrocytes, and leukocytes.

### Platelets

Platelets contribute to the thrombotic risk, as suggested by the observation that cytoreductive therapy with hydroxyurea reduces the incidence of thrombosis[9]. A recently published analysis by Marchioli et al.[6] reported that antiplatelet therapy was significantly associated with a lower risk of cardiovascular events. However, it is not clear whether the benefit of cytoreductive therapy is due to platelet count reduction *per se* or the overall effect of myelosuppressive agents upon factors including hematocrit and white cell count.

Platelet activation and thrombotic risk in MPN has also been investigated. Increased expression of P-selectin, thrombospondin, and the activated fibrinogen receptor GPIIb/IIIa by platelets has been found to correlate with thrombosis[10,11]. Formation of platelet microparticles associated with the expression of platelet procoagulant activity has been found to correlate with thrombosis in PV and ET[12].

## Platelet Receptors and Activation

Whether the quantity and quality of receptors expressed on the platelet surface may affect the thrombotic risk in patients with MPN is unclear. The fact that a high variability in platelet receptor expression has also been observed in normal subjects makes the evaluation of this topic problematic.

The frequency of the polymorphisms of platelet adhesion molecules GPIb[alpha], GPIIIa, and GPIa in patients with PV or ET has been previously evaluated. In particular, the frequencies of the polymorphic variants of a gene were compared between patients who did or did not have thrombotic complications. This study found a statistically significant association between the frequency of the $Pl^{A2}$ allele of GPIIIa and the frequency of arterial thrombosis in patients with PV[13].

Enhanced platelet activation has already been demonstrated in patients with PV and ET. Activated platelets interact with other blood components, both cellular and circulating, and have the capacity to provoke endothelial activation/damage. A correlation has been shown between thrombosis and features of platelet activation, such as P-selectin and thrombospondin. Platelet microparticles have also been linked to platelet activation and procoagulant activity[12].

A prospective study investigated platelet function by simultaneous measurement of platelet aggregation and ATP-dense granule release by whole blood platelet lumi-aggregometry. These authors found a high prevalence of *in vitro* platelet hyperactivity in MPN patients with a previous history of thrombosis and suggested that this functional test may efficiently select MPN patients for aspirin therapy[14].

## Leukocyte

Most patients with PV and ET typically present with leukocytosis, which has been shown to play a role in the pathogenesis of a thrombophilic state. In fact, there is evidence of enhanced thrombotic risk in patients with vascular disorder and high leukocyte count[15,16]. Recently published studies also identify leukocytosis as a potential risk factor for thrombosis in patients with PV and ET[17]. The pathogenetic mechanism responsible for the thrombophilic state in PV and ET patients with leukocytosis has also been investigated. Activated neutrophils may favor an imbalance toward a prothrombotic state by different mechanisms. Proteases released by neutrophils, such as elastase and cathepsin G, may directly damage the endothelium[18,19]. Falanga et al. studied the interplay between neutrophils, endothelium, and coagulation in PV and ET patients. These authors were able to demonstrate that the presence of circulating activated neutrophils correlated with plasma markers, reflecting both activation of coagulation and perturbation of the endothelium[20]. Since clinical data indicate an association of the JAK2V617F mutation with the severity of the MPN disease, it is of interest to understand whether the mutation status may specifically affect the hemostatic system[21]. A study from our group investigated the expression of procoagulant, adhesive, and inflammatory molecules by platelets and neutrophils of ET patients, as well as plasma markers of hemostatic activation, according to the presence of the JAK2V617F mutation[22]. The results showed an elevated expression of platelet surface tissue factor (TF) in the JAK2V617F carriers compared to wild-type subjects. The surface neutrophil activation/inflammatory markers (i.e., CD14, TF, CD11b, and leukocyte alkaline phosphatase [LAP]) were all significantly higher in the whole ET group vs. controls and, in particular, CD14 and LAP were demonstrated to be more highly expressed in JAK2V617F mutation carriers. The greater neutrophil activation occurring in JAK2V617F mutation carriers could render ET patients more susceptible to vascular damage. Recently, our group demonstrated in PV and ET patients an involvement of both the JAK2 mutation and leukocyte activation in determining the occurrence of an acquired activated protein C resistance phenotype, as shown by the thrombin generation assay[23].

## Platelets-Leukocytes Interaction

Activated neutrophils and platelets can interact to generate neutrophil-platelet mixed aggregates. Increased levels of these aggregates have been found in several pathological conditions associated with a propensity to thrombosis[24,25]. The interaction between platelets and neutrophils occurs by several cell membrane adhesion molecules, including platelet P-selectin (CD62P) binding to P-selectin glycoprotein ligand-1 (PSGL-1) on neutrophils. Falanga and colleagues recently determined levels of platelet-neutrophil aggregates in a cohort of PV and ET patients[26]. They found that neutrophil-platelet aggregates were increased in PV and ET patients, mainly in JAK2V617F-positive patients as compared to JAK2V617F-negative patients, and that patients on aspirin had significantly reduced aggregate formation.

## Endothelium

Physiologically, the endothelium facilitates blood flow by providing an antithrombotic surface that inhibits platelet adhesion and activation of coagulation. Several factors may affect endothelium function in patients with MPN.

The activation of platelets and leukocytes observed in PV and ET may perturb the resting state of the endothelium and turn it into a surface triggering a prothrombotic status. Activated neutrophils release reactive oxygen species and intracellular proteases, which can act on endothelial cells modifying the hemostatic balance toward a prothrombotic state. Proteases can induce detachment or lysis of endothelial cells affecting functions involved in thromboregulation[18]. Importantly, it has been demonstrated that damage of the endothelium determines the release into the circulation of specific markers, such as vWF. This is of particular relevance in the pathogenesis of thrombosis in MPN as, once platelets bind to vWF, they are activated and then able to aggregate and strengthen the clot. This mechanism may partly concur with the observed high incidence of thrombosis in patients with MPN[20,27,28].

Other plasma markers of endothelial, platelet, and leukocyte activation have been studied with the aim to better define the risk of thrombosis in patients with PV and ET.

## Selectins

Selectins constitute a family of adhesion molecules expressed by the endothelium (P-selectin and E-selectin), platelets (P-selectin), and leukocytes (L-selectin)[29]. As they are released into the circulation, their detection has been used as indices of endothelial, platelet, and leukocyte activation. High levels of membrane-bound and soluble plasma P- and E-selectin have been found in ET patients with thrombosis, suggesting that sustained endothelium and platelet activation in PV and ET patients might contribute to the pathogenesis of thrombosis[30,31].

We have recently studied soluble plasma selectins in patients with MPN[32]. We found increased levels of soluble P-, E-, and L-selectins in PV and ET patients compared to controls. In particular, PV patients showed higher levels of P-selectin as well as E-selectin, both possibly released from activated endothelium. These findings further support the hypothesis of the occurrence of endothelial perturbation in these patients.

## Nitric Oxide

In addition to releasing substances that stimulate thrombus generation following injury, endothelial cells and activated platelets release the platelet inhibitor nitric oxide (NO), providing a negative feedback mechanism for the propagation of thrombus formation[33]. NO is a free radical product generated through the oxidation of L-arginine to L-citrulline by NO synthases (NOS). The endothelial-derived NO

is one of the main mediators influencing vascular hemodynamics and the interaction of leukocytes and platelets with endothelial cells. In fact, NO mediates vascular relaxation in response to vasoactive substances and shear stress; inhibits platelet adhesion, activation, secretion, and aggregation; and promotes platelet disaggregation. Moreover, NO inhibits expression of P-selectin on platelets as well as it impairs leukocyte adhesion to the endothelium.

Clinical conditions have been reported in which a deficiency of endogenous NO production may contribute to a thrombotic event[33]. For example, impaired platelet-derived NO may contribute to the development of acute coronary syndromes by influencing platelet function or recruitment and consequent thrombus formation[34].

Our group recently studied circulating NO in MPN patients[32]. We found reduced plasma levels of NO in patients with ET compared to controls. This finding confirms previous observations that in MPN patients with thrombocytosis, the production of NO by platelets is impaired[35]. However, in the same study and for the first time, we observed that ET patients treated with hydroxyurea presented with the highest levels of plasma NO. A similar effect of hydroxyurea on NO plasma levels has been reported in patients with sickle cell anemia[36] and it may contribute to the well-known ability of hydroxyurea to prevent thromboembolic complications in ET patients[37]

In the same study, PV patients showed high plasma NO levels compared to controls and these levels were not affected by hydroxyurea treatment. This is not unexpected as it has been shown that high hematocrit levels are associated with increased NO levels in the blood[38]. This may represent a compensatory mechanism in a situation of high thrombotic risk.

## SUMMARY

Current treatment of PV and ET is mainly aimed at preventing major cardiovascular events and is based on the risk category definition. Age and thrombotic history represent the most important risk factors for thrombosis in PV and ET patients, as was consistently demonstrated by several studies[6,39,40]. However, as discussed in this review, other novel disease-associated risk factors have generated interest with regard to their inclusion in the evaluation of the thrombotic risk in MPN patients.

Leukocytosis and the JAK2V617F mutation have the strongest rationale based on preliminary data, and they certainly deserve validation in clinical trials. Of interest, these two factors may act independently or in association in triggering a hypercoagulable condition in patients with MPN. It would be of utmost importance to define the impact of these factors, particularly in low-risk patients, as this subgroup may potentially benefit from treatment with novel drugs such as anti-JAK2 agents or cytoreductive therapy.

Future clinical trials should also address the risk of thrombotic recurrence in PV and ET patients with leukocytosis and/or the JAK2V617F mutation. In fact, interesting preliminary data are available, suggesting that cytoreductive therapy may be protective in reducing the incidence of recurrent events in PV and ET patients with arterial thrombosis, although this observation has been not confirmed by others[41,42].

In conclusion, current knowledge on the incidence of thrombosis in patients with PV and ET is encouraging, but still inadequate for reliably estimating the risk. Additional information is clearly needed and hopefully will become available from future large-scale clinical studies.

## ACKNOWLEDGMENTS

The studies conducted in Dr. Falanga’s laboratory were partly supported by a grant from the “Associazione Italiana per la Ricerca sul Cancro (AIRC)”.
